# SUITABLE? THE RHETORICAL AND LIFE COURSE IMPLICATIONS OF SERENA WILLIAMS AND THE CATSUIT

**DOI:** 10.1093/geroni/igz038.1972

**Published:** 2019-11-08

**Authors:** Brianne M Stanback

**Affiliations:** University of South Florida, Tampa, Florida, United States

## Abstract

Rhetorical inquires have shown connections between representation and power, workplace fashion and development of ethos, and the rhetoric of glamour through women’s fashion and dress. One element absent from that conversation is how the life course, which typically differs for women because of existing power structures advantaging men, may impact the experience of women as they age, their choice of dress, and the rhetorical implications of those decisions. To explore dress and rhetoric from a life course perspective, this project traces the evolution of Serena Williams’ work apparel across her professional tennis career to the catsuit worn at the 2018 French Open, which is the focus of the project. Press reports on the 2018 catsuit by Nike, New York Times, Sports Illustrated, Business Insider, BBC Sport, Washington Post, and Los Angeles Times, interviews given by Williams, and the television documentary, Becoming Serena, will be analyzed for their treatment of Williams’ work attire and the life course. Responses to the catsuit emphasize attitudes about gender, race, and class, either discounting or ignoring the life course implications such as motherhood and changes in health status. Despite professional success, responses about the catsuit may reflect that Williams faces the same jeopardies, and invisibility, common to many women as they age, and the rhetorical perspective provides new methodological and pedagogical possibilities for instruction in aging.

